# Magnitude and factors associated with preoperative depression among elective surgical patients at University of Gondar comprehensive specialized hospital, North West Ethiopia: A cross-sectional study

**DOI:** 10.1016/j.amsu.2022.103341

**Published:** 2022-02-10

**Authors:** Yeneneh Negesse Kebede, Zewditu Abdissa Denu, Habtu Adane Aytolign, Abraham Tarekegn Mersha

**Affiliations:** a Department of Anesthesia, Debre-Tabor Referral Hospital, Amhara National Regional State, Ethiopia; b Department of Anesthesia, School of Medicine, College of Medicine and Health Science University of Gondar, Gondar, Ethiopia

**Keywords:** Depression, Preoperative, Magnitude, Associated factors, ASA, American Society of Anesthesiologists, BMI, Body Mass Index, DM, Diabetes Mellitus, IHD, Ischemic Heart Disease, PHQ-9, Patient Health questioner-9, UGSCH, University of Gondar comprehensive specialized hospital, USA, United States of America

## Abstract

**Background:**

Depression one of the world's prevalent mental illnesses is a leading cause of major public health problems globally and its frequency has been increasing, particularly in low and middle-income countries. Little is known about the magnitude and contributing factors of preoperative depression among elective surgical inpatients in the country and in the study area as well. The aim of the current study was to assess the magnitude and factors associated with preoperative depression among elective surgical inpatients.

**Method:**

A cross-sectional study was conducted from May 01, 2021 to June 30, 2021 among preoperative surgical inpatients at University of Gondar comprehensive specialized hospital. Non probability sampling was used. A nine-item questionnaire screening tool was used to assess depression. We computed the bi-variable and multivariable binary logistic regression analyses. Crude and adjusted odds ratio with 95% confidence interval were used.

**Result:**

The magnitude of depression was 28.3%. In the multivariable logistic regression analysis female (AOR = 2.27, 95% CI: 1.15, 4.5), being widowed (AOR = 3.271, 95% CI: 1.25, 8.56), divorced (AOR = 3.41, 95% CI: 1.13, 10.26), length of hospital stay of 7–14 days (AOR = 2.7, 95%CI: 1, 7.2) and more than 14 days (AOR = 3.19, 95% CI: 1.3, 7.8), having co-existing diseases (AOR = 2.78, 95%CI: 1.28, 6.02), current history of pain (AOR = 3.12, 95%CI: 1.6, 5.7), admission to orthopedics (AOR = 3.28, 95%CI: 1.55, 6.95) and gynecology ward (AOR = 2.43, 95% CI: 1.03, 5.7) and poor social support AOR = 2.24, 95% CI: 1.1, 4.6) were significantly associated with depression.

**Conclusion:**

The magnitude of pre-operation depression was 28.3%. Female, Widowed, being divorced, length of hospital stays, coexisting chronic illness, current history of pain, admission at orthopedic and gynecology wards and poor social support were factors significantly associated with depression. We recommend strengthening the linkage of the psychiatric department with preoperative patients to provide psychotherapy behavioral modification.

## Highlights

• Depression is one of the world's mental illnesses problem.
• Little is known about prevalence and its contributing factors of preoperative depression.
• Surgical patients with Preoperative depression have major post-operative complications.
• The prevalence of preoperative depression among surgical inpatient was 28.3%.

## 1 Introduction

Depression is a common mental disorder in the world which is characterized by despair and sadness, difficulties in thinking and decision making, loss of interests and pleasure in normal, enjoyable things accompanied by an inability to carry out daily functions, appetite, sleep and psychomotor disturbances and suicidal ideation [1,2]. In the global arena, depression is one in the fourth, leading causes of diseases representing 12% of disability [3].

Moreover, admission to hospital may lead to depression as it disrupts usual life and there is the prospect of medical or surgical interventions as well as loss of independence and body image [4,5]. In addition, anticipated surgery and anesthesia is the major stress factors for patients scheduled for surgery [6].

Although depression is common and associated with a high burden due to disability and mortality, only a small percentage are recognized when it comes to patients awaiting surgical interventions. Moreover, under detection and under treatment of depression continues to be a serious [7]. Reason of under recognition and treatment of depression in patients seeking a surgical intervention and its impact on surgical and anesthetic management outcomes still remain unknown [8]. Even screened in the preoperative period, about 71% depressed patients do not get treatment [9].

In the world, the magnitude of preoperative depression ranges from 11% to 88% [5]. This is quite staggering which in turn causes a speculation about the contribution of uncovered surgical or anesthetic risk factor for this big gap to exist.

Surgical patients with Preoperative depression have major post-operative complications. Some of them are longer hospital stay [10], decreased immunity [11] leading to increased post-operative surgical infection [12], preoperative medication omission [13], poor functional recovery [14] and decreases post-operative acute pain threshold [15,16] which further increase risk of chronic pain and associated complications. Therefore, depression should be screened and treated before surgical intervention. Data on depression in preoperative patients in the country are scarce, particularly in the study area.

Therefore, the aim of this study was to investigate the magnitude and associated factors with preoperative depression among scheduled elective surgical inpatients.

## 2 Methods

### 2.1 Study design and setting

Institution-based cross-sectional study was conducted from May 01, 2021 to June 30, 2021. The study was conducted at University of Gondar comprehensive, specialized hospital. This hospital undergone a wide variety of surgical procedures, both emergency and elective, most commonly, gynecology and obstetrics, general surgery including, pediatrics, trauma and orthopedics and ophthalmology. This study was conducted in general surgical, orthopedic, gynecology and ophthalmology wards in patients who were scheduled for surgery. Those elective patients are scheduled for surgery before two days of the actual surgical procedures on the operation room schedule board for both surgical and anesthesia evaluation, preparation and optimization. The anesthesia evaluations are performed after the schedule and those patients who are not fit for anesthesia and surgery are informed of them and optimized for another day depending on the severity of illness. After ethical clearance was obtained from ethical review committee and informed consent from the participant, depression was assessed during the anesthesia evaluation period on those scheduled surgical inpatients.

The research is registered with unique identifying number of researchregistry7471 and reported in line with STROCSS 2021 criteria [17].

### 2.2 Study population

All General surgical, Orthopedic, Gynecological and Ophthalmic patients who were scheduled for surgery during study period were study population. All patients in surgical, orthopedic, gynecologic and ophthalmic ward, aged ≥18 years who were scheduled for elective surgery were included. Whereas, known diagnosed psychiatric patients or on medication of antipsychotic drugs, patients who were mentally handicapped, those refusing to participate and who do not understand Amharic or English languages were excluded.

### 2.3 Variables

#### 2.3.1 The outcome variable of the study was depression

The independent variables were Socio-demographic, Social and Substance-Related Factors, previous and current clinical condition.

### 2.4 Sample size determination and sampling procedure

The single population proportion formula was used to determine the sample size, since there was no previous study done similar to this in low-income countries, so that we took proportion of 50% by assuming 95% of confidence interval, 5% margin of error, and finally the sample size was calculated as:

$n=\;\frac{\left(z_{\frac{a}{2}}\right)2\;p\left(1-p\right)}{e^{2}}$$n=\;\frac{1.96^{2}\times 0\;.5\times 0.5\;}{0.05^{2}}$**,** n = 384, Where p = 50% from p = 50% rule, z = 1.96, and e = 0.05.

After adding 10% of non-response rate, the final sample size was 424.

All consecutive patients who fulfilled the inclusion criteria were used until the required sample achieved.

### 2.5 Operational definition

Based on Patient Health Questionnaire- 9 (PHQ -9) [18,19].

Minimal depression: PHQ-9 score 1-4.

Mild depression: PHQ-9 score 5-9.

Moderate depression: PHQ-9 score 10-14.

Moderately severe depression: PHQ-9score 15-19.

Severe depression: PHQ-9 score 20-27.

Substance use history: A participant who ever has a history alcohol drinking, Khat chewing and cigarette smoking [20].

Chronic illness: Presence of one or more diseases among hypertension, heart disease, cancer, diabetes, or any clinically diagnosed disease [21].

Length of hospital stay: duration of admission to the hospital till discharge [22].

### 2.6 Instrument, data collection procedure and quality management

Social support was assessed using the Oslo 3-item social support scale. It measures the levels of social support, including the number of people feel close to the participants, the interest and concerns showed by others as well as the ease of obtaining practical help from others. The sum score scale is from 3 to 14 and has three classifications: “poor social support” 3–8, “moderate support” 9–11, and “strong support” 12–14 [23]. It has been used in Ethiopia in a variety of clinical settings [24].

We used Patient Health Questionnaire 9 to assess depression. A PHQ-9 is a 9-item questionnaire, commonly used to screen for symptoms of depression in hospital admitted patients. It is translated to Amharic language and validated with the sensitivity 86% and specificity 67%. A day training was given for data collectors and supervision was undertaken on daily bases. Data were collected by two delegated data collectors using pretested interviewer administered questionnaire. When patients were found to be depressed during data collection, the data collector informed the in charge surgical team for further diagnosis and the possible tailored interventions according to the patients’ clinical need.

### 2.7 Data processing and analysis procedures

The data were entered into EpiData version 4.6 and imported to SPSS version 26. Descriptive statistics was done to identify the distribution of sociodemographic, clinical and behavioral characteristics of the study participants. Bivariable and multivariable logistic regression and odds ratio with 95% were used. The Variables with p-value < 0.05 were used as the cutoff point and independent variables with p-value ≤ 0.2 in bivariate logistic analysis was fitted into multi variable logistic regression to identify independently associated factors in the final model.

## 3 Results

### 3.1 Socio-demographic characteristics

Out of 422 study participants, 420 were involved in this study with the response rate of 99.5%. The mean age of the study participants was 42.17 ± 14.41 years and more than half (55.5%) of them were male. About (37.86%) of them were found to be both unable to read and write (Table 1). Regarding with occupation, majority were farmers (32.86%) and (25.24%) housewives (Table 1). While the majority were married (65.24%) (Table 1). Most of the participants (63.8%) were living in the rural areas (Table 1).

**Table 1:** Distributions of sociodemographic factors in preoperative surgical inpatients, 2021 (n = 420).

Variables	Category	Frequency	Percent (%)
Sex	Male	233	55.48
	Female	187	44.52
Marital status	Married	270	64.29
	Single	94	22.38
	Divorced	21	5
	Widow/widower	35	8.33
Residency	Urban	150	35.71
	Rural	270	64.29
Ethnicity	Amhara	399	95
	Tigre	10	2.38
	Oromo	11	2.62
Religion	Orthodox	370	88.09
	Muslim	30	7.14
	Protestant	16	3.81
	Catholic	4	0.96
Educational level	Unable read/write	159	37.86
	Able read/write	67	15.95
	Primary	54	12.86
	Secondary	65	15.48
	Diploma, degree, or above	75	17.86
Occupations	Employed	72	17.14
	Private	73	17.38
	Farmer	138	32.86
	Housewife	106	25.24
	Daily worker	31	7.38
Living alone	Yes	398	94.76
	No	22	5.24

### 3.2 Clinical characteristics

Of the 420 participants, majority (57.1%) was admitted to the surgical ward (Table 2). Moreover, out of the total numbers 63 (15%) had coexisting chronic illness and 36 (8.6%) had previous history of hospital admission (Table 2).

**Table 2:** Distribution of clinical factors in preoperative surgical patients, 2021 (n = 420).

Variables	Category	Frequency	Percent (%)
ASA class	Class 1	286	68.10
	Class 2	118	28.10
	Class 3	16	3.80
Current history of pain	No	335	77.4
	Yes	95	22.6
History of chronic illness	No	357	85.00
	Yes	63	15.00
Patient or Family history of psychiatric disorder	No	385	91.7
	Yes	35	8.3
Length of hospital stay	Less than 1 week	344	81.90
	Up to 2 weeks	30	7.10
	More than 2 weeks	46	11.00
Previous anesthetic and surgical exposure	No	67	16
	Yes	357	84
Type of anesthesia planned	General	252	60.00
	Regional	168	40.00
Physical activity	No	106	74.80
	Yes	314	25.20
Previous hospital admission	No	389	91.00
	Yes	38	9.00

### 3.3 Social and Substance-Related Factors

Among the total of the participants (19.8%) them found to have poor social support. Whereas, about (7.38%) of the respondents had a history of lifetime alcohol consumption (Table 3). While 8.81% and 11.66% of them were previous chat addicted and smoker respectively (Table 3).

**Table 3:** Distributions of Psychosocial characters and substance use in the preoperative surgical inpatients, 2021 (n = 420).

Variables	Category	Frequency	Percent (%)
Previous alcohol use	Yes	116	27.67
	No	304	72.38
Current alcohol use	Yes	203	48.33
	No	217	51.67
Previous khat use	Yes	37	8.81
	No	387	91.9
Current khat use	Yes	23	5.48
	No	399	94.52
Previous cigarette smoking	Yes	49	11.66
	No	379	88.34
Current cigarette smoking	Yes	26	6.19
	No	394	93.81
Current and Previous other addictive substance use	Yes	8	1.43
	No	412	98
Social support	Poor	83	19.76
	Moderate	118	28.09
	Strong	219	52.14

### 3.4 The magnitude of depression

**Fig. 1:**
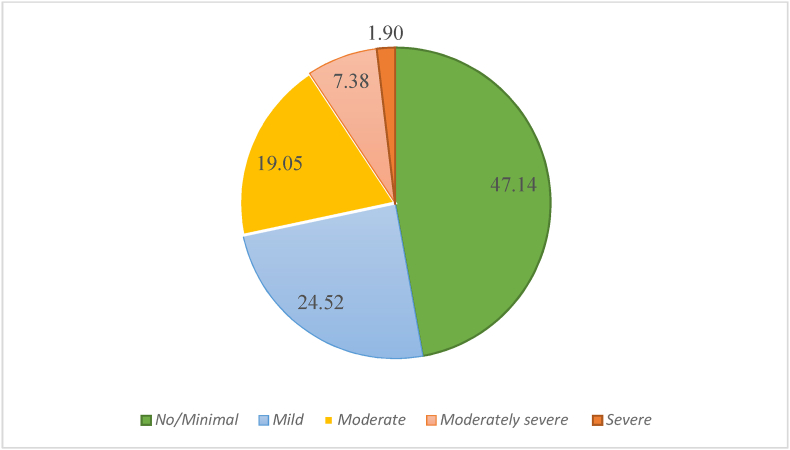
Magnitude of Depression among preoperative surgical inpatients, 2021 (n = 420).

The magnitude of preoperative depression was 28.3% (95% CI 23.7, 32.7). Moreover, the severity of preoperative depression is seen below (Fig. 1).

### 3.5 Factors associated with depression

Of the independent variables sex, widowed, divorced, length of hospital stay, coexisting of chronic illness, history of pain, wards admitted to, the level of social support, previous history of hospital admission, ASA status, the level of education, history of psychiatric illness, occupation, residency and age yielded p-value ≤ 0.2 in the bivariable logistic regression and were considered in the multivariate logistic regression model.

The multivariate analysis (Table 4) showed that being female were more than two times likely to develop depression (Table 4). Similarly, being either divorced (AOR = 3.41, 95% CI:1.13, 10.26) or widowed (AOR = 3.271, 95% CI:1.25, 8.56) were approximately three and half times more likely to lead to depression when compared to being married. The odds of developing depression was three times higher among those respondents who stayed in the hospital more than two weeks as compared to those who stayed less than one week (AOR = 3.19, 95% CI:1.3, 7.8). Depression occurred 2.8 times more in the presence of coexisting chronic illness (AOR = 2.78, 95%CI:1.28, 6.02). History of current pain was about 3 times increased the risk of depression compared pain free patients (AOR = 3.12, 95%CI: 1.6, 5.7). Being admitted to orthopedics and gynecology ward was 3.3 times to develop depression compared to the general surgical ward (AOR = 3.28, 95%CI: 1.55, 6.95), and being admitted to gynecology ward found to be 2.4 times having depression (AOR = 2.43, 95% CI: 1.03, 5.7) (Table 4). Participants who had poor social support were over two times more likely to lead to depression as compared to respondents who had strong social support (AOR = 2.24, 95% CI: 1.1, 4.6) (Table 4).

**Table 4:** Bivariable and multivariable analyses among preoperative surgical inpatients, 2021 (n = 420).

Variables		depression		COR (95% CI)	AOR (95% CI)	p-value
		Yes	No			
Sex	Male	51	182	1	1	
	Female	68	119	2.04(1.33, 3.14)	2.27(1.15, 4.5)	0.018
Marital status	Married	67	211	1	1	
	Widow/widower	20	15	4.77(2.3, 9.89)	3.27(1.25, 8.56)	0.016
	Divorced	13	8	5.81(2.30, 14.68)	3.41(1.13,10.26)	0.029
Length of hospital stay	≤7 days	74	270	1	1	
	8–14 days stay	14	16	4.17(1.95, 8.93)	2.7(1, 7.2)	0.048
	>14 days stay	29	17	6.22(3.24, 11.94)	3.19(1.3, 7.8)	0.011
Coexisting chronic illness	No	29	272	1	1	
	Yes	29	34	3.75(2.16, 6.52)	2.78(1.28, 6.02)	0.009
History of current pain	No	45	256	1	1	
	Yes	50	45	4.12(2.54, 6.68)	3.07 (1.6, 5.7)	0.001
Wards admitted to	General Surgery	48	201	1	1	
	orthopedics	47	46	4.28(2.56, 7.15)	3.28(1.55, 6.95)	0.0018
	gynecology	21	34	2.57(1.38, 4.83)	2.42(1.03, 5.7)	0.043
Level of social support	Strong	56	163	1	1	
	poor	34	49	2.012(1.19, 3.44)	2.24(1.1, 4.6)	0.028

## 4 Discussion

This study was carried out to screen preoperative depression symptoms and associated factors among elective surgical inpatients. The magnitude of depression in our result is in line with studies carried out in hospitalized patients in Iran (29.5%) [25] and it also matches with studies conducted by Walker, J et al. among hospital inpatients (5%–34%) [26]. The other two studies conducted among, Brazilian (28%) and Germany (29.7%) had approximately the same magnitude of depression with our study result [27,28]. On the contrary, the magnitude of depression in our study is higher than those of other studies done among surgical patients in Germany (11.3%) [10], Norway (13%) [9], and Italy (21%). The reason might be sociocultural variation, difference site of ward admitted, type of patents and difference study population. However, the magnitude of depression in our study was quite low compared to studies conducted among hospital admitted patients [[29], [30], [31]]. The variation in the above rates might be due to age of participants, the use of various scales and rating for assessing the level of depression and types of admission site.

In this study; female was significantly associated with depression, which is supported by studies done in the country among admitted surgical and medical patients, in Brazil and Iran [[31], [32], [33]]. The possible reasons might be some mood changes and depressed feelings occur with normal hormonal changes. Furthermore, there are different factors that may increase the risk of depression symptoms in females such as biological factors and sociocultural factors. The Female may face some form of trauma during their lifetime like gender violence, discrimination, leading to high anxiety, social withdrawal, and low self-confidence which in turn increased depression symptoms.

Being a widower or widowed and divorced was greater than three times more likely to have depression. This was consistent with other studies carried out in other parts [30,31], china [34] and Uganda [35]. The possible explanation might be Divorce and widowed are associated with significant risk of emotional distress, including clinically-significant psychological depression the reason might also be the sociocultural attitude and norms of less respecting the divorced and widowed, being single has also psychological impact, probability of losing the next chance of marriage.

This study pin outed that the length of hospital stay was significantly associated with depression in the preoperative period. Study findings in Iran and Germany conducted among surgical patients and patients in the preoperative anesthesia assessment clinic respectively go in line with ours [25,33]. The same results were found in China [36] and Israel [37]. Hospital stay affects the mind and mood, the environment is stressful with a variety of patients having different disease, feeling unwell of the probability of being infected with another hospital acquired disease. Feeling confused of their resources consumed in the hospital.

Another factor associated with depression in our study was coexisting chronic illness. This finding is supported by studies [31]. Might be the illness affects their quality of life and the side effect of drugs, the outcome of surgery in combination of illness.

Patients with complaint of pain were associated risk of depression, which is in line with the result of study in Boston, USA [38]. The possible reason could be that pain is unpleasant sensory and emotional experience associated with actual or potential tissue damage which might increase depression symptoms.

In this study being admitted to orthopedic and gynecology wards were factors significantly associated with depression. The possible reason why we found this could be patients admitted to the orthopedic ward probably had pain, disability, fracture and thinking of future outcome after surgery. Gynecology patients are naturally females, some mood changes and depressed feelings occur with normal hormonal changes. Furthermore, most of gynecology ward patients were with the diagnosis of fistula and gynecologic tumor.

Respondents with history of poor social support, in our study were another significant factor to develop depression. This result was supported by other studies in Berlin [30] and Germany [39]. The possible explanation could be being scheduled for surgery is stressful condition in which they might think of morbidity and mortality so, patients need strong social support both in psychological makeup and resource.

## 5 Strength and limitations of the study

As per our knowledge, it might be the first study conducted among the preoperative surgical patients in the country. It might have also limitations, such as the study design, which was cross-sectional, making it difficult to conclude that the observed associations were necessarily causal in nature.

## 6 Conclusion and Recommendations

The magnitude of depression symptoms among the preoperative surgical inpatients was 28.3%. Depression was significantly associated with being female, being divorced, widow/widower, coexisting diseases, current history of pain, ward admitted to and poor social support. We recommend that strengthening the linkage of the psychiatric department with preoperative patients to provide psychotherapy, behavioral modification which may help them to avoid negative feeling. Thus, screening and treating before surgical intervention may have better outcomes, especially the above-mentioned associated factors need counseling and behavioral modification. Moreover, we recommend the surgical team to do operation as early as possible to avoid length of hospital stay.

## Availability of data and material

All data of the current study are available on reasonable request.

## Competing interest

All authors declared that they have no any competing interests.

Research registration with UIN: researchregistry7471.

Hyperlink to your specific registration (must be publicly accessible and will be checked): https://www.researchregistry.com/browse-the-registry#home/

## Provenance and peer review

Not commissioned, externally peer-reviewed.

## Ethical approval and consent to participate

Ethical clearance was obtained from University of Gondar College of Medicine and Health Science, School of Medicine Ethical Review Committee. Written and verbal informed consent was obtained from each study participant after clear explanation about the merits of the study.

There was no incentive or payment in cash or in kind to be gained by taking part in this project. Patients who were not willing to participate in the study were informed that, they had full right not to participate or stop at any time. Confidentiality was guaranteed by keeping the concealment of personal identification, keeping completed questionnaires and results in well-secured area.

## Ethical approval

Ethical clearance was obtained from the ethical review committee of the school of medicine. Official permission letter was obtained to conduct research from the University of Gondar comprehensive specialized hospital administrator. Written consent was taken from all study subjects and to secure the confidentiality of patient information. Anyone not willing to participate in the study was informed that, they had full right not to participate or stop at any time. If patients were in depression during this study period, either the data collectors informed to the responsible health professional to treat them. Confidentiality was also be guaranteed by keeping the secrecy of personal identification, keeping completed questionnaires and results in well-secured area.

## Sources of funding

Amhara Regional Health Bureau.

## Author contribution

All authors (YN, ZA, HA, and AT) participated in the conception and design of the study. YN performed data entry, analysis and drafted the manuscript. All authors (YN, ZA, HA and AT) approved the final work. All authors critically revised the manuscript and have approved the final version.

## Registration of research studies

Name of the registry: research registry.

Unique Identifying number or registration: researchregistry7471.

Hyperlink to your specific registration (must be publicly accessible and will be checked): https://www.researchregistry.com/browse-the-registry#home/

## Guarantor

habituadane@gmail.com.

Phone number:+251930699881.

p.o.box:196.

## Consent

Not applicable.

## Funding

Amhara Regional Health Bureau for data collection.

## Declaration of competing interest

There is no conflicts of interest.
